# Supplementary material for: An artificial chromosome ylAC enables efficient assembly of multiple genes in Yarrowia lipolytica for biomanufacturing
Source: Commun Biol. 2020 Apr 29;3:199. doi: 10.1038/s42003-020-0936-y (PMC7190667; doi:10.1038/s42003-020-0936-y)
Supplement: Supplementary file 1 — Supplementary Information [file 42003_2020_936_MOESM1_ESM.pdf]

Supplementary Figures

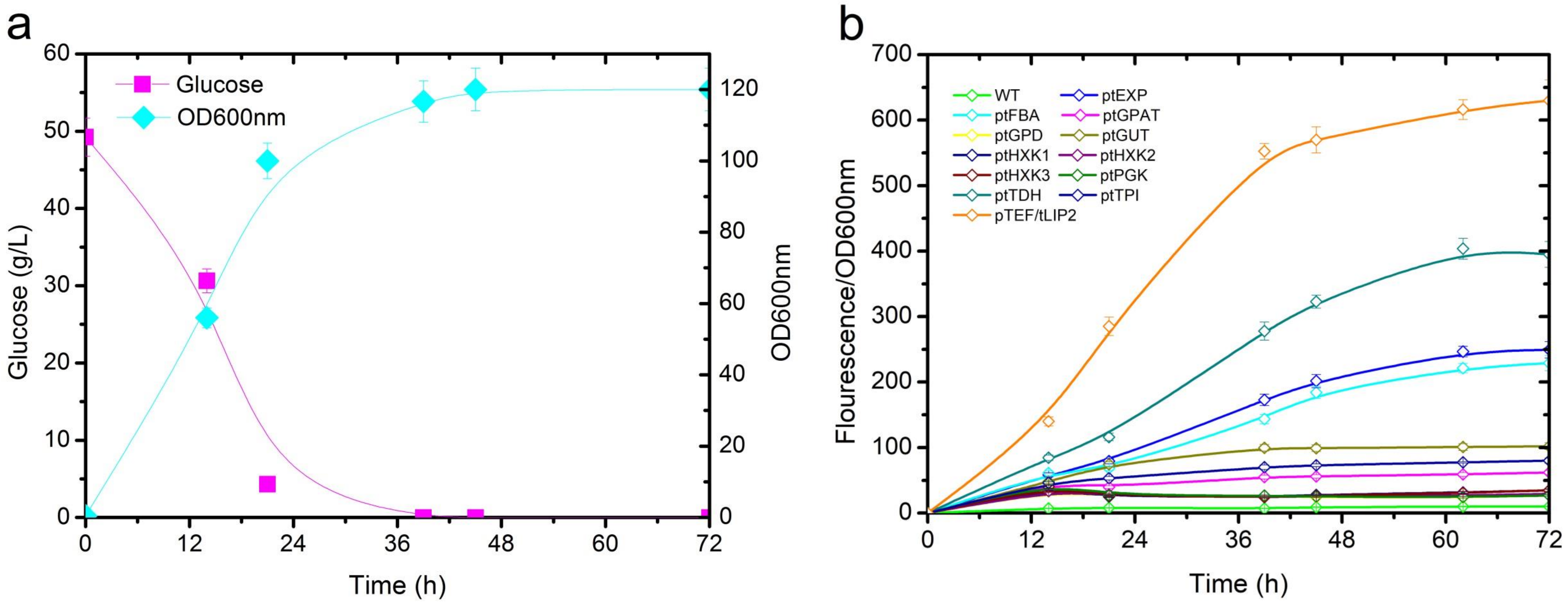

Supplementary Figure 1 Production of DsRed under the control of different promoters and terminators in *Y. lipolytica.* Shown are: (a) the representative growth curve and the substrate consumption profile for the transformants and the wild type stain; (b) the intensity of fluorescence normalized by OD of the *Y. lipolytica* transformants during cell cultures in YPD. The results were calculated from at least five biological replicates, error bars are standard deviation.

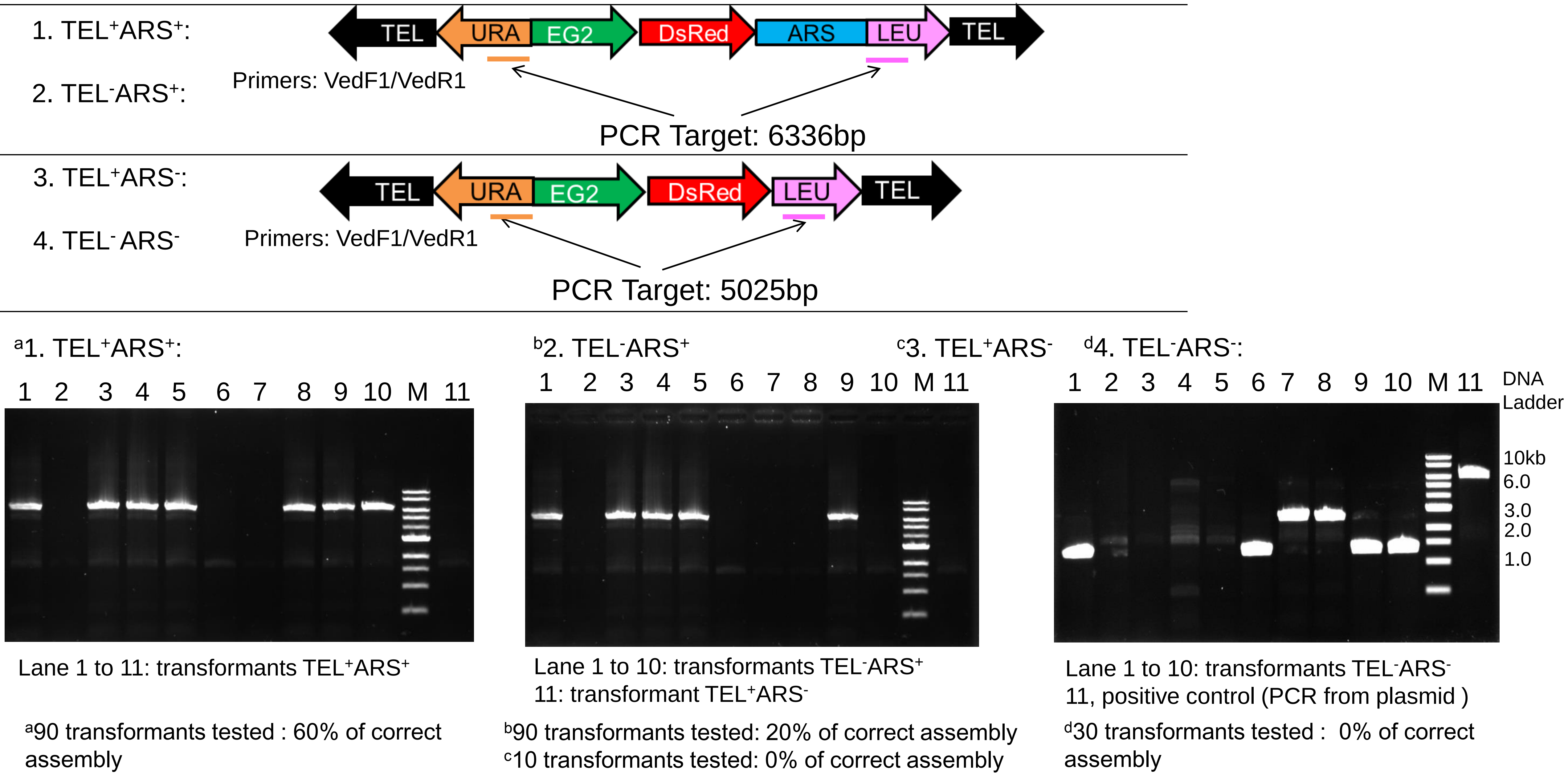

Supplementary Figure 2 PCR verification of assembling of ylAC and reporter genes.

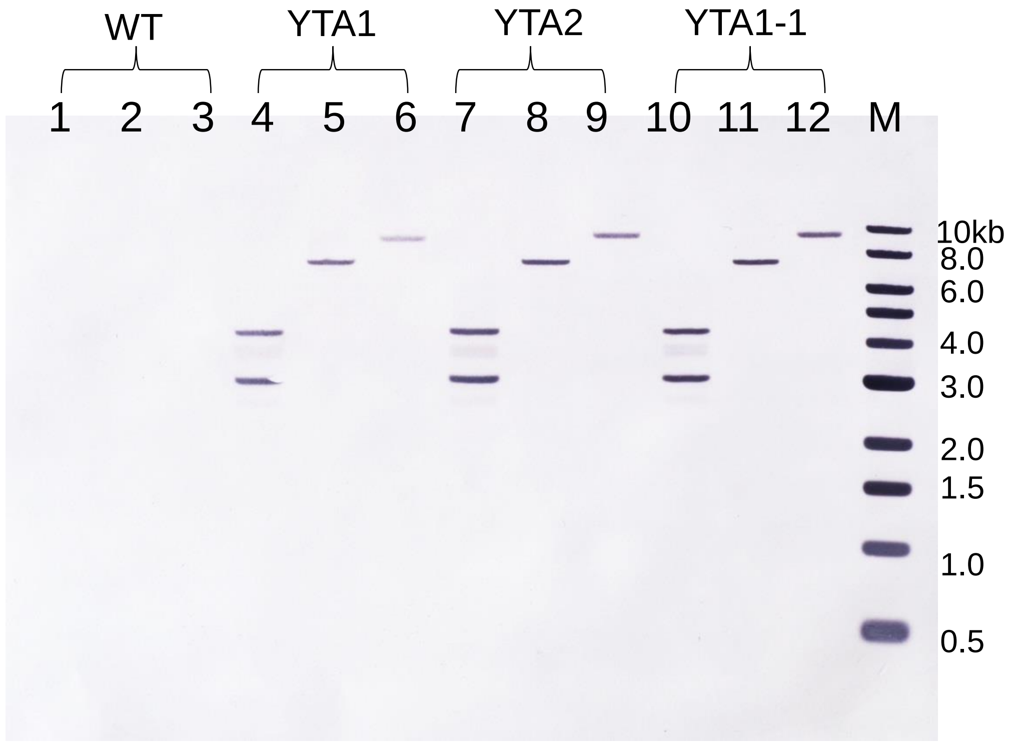

Supplementary Figure 3 Southern blot analysis of the gDNA of the wild type strain (WT), the initial transformant (YTA1), and *Y. lipolytica* re-transformed with the linear DNA fragment isolated from YTA1 (YTA1-1, 2 and 3). The *RedStar2* gene was used as the target for a specific probe to detect gDNA fragments generated by restriction enzyme digestion. Lanes 1, 4, 7 and: gDNA digested by *Cla*I/*Nde*I (expected sizes: 4494 and 2949 bp), lanes 2, 5, 8: gDNA digested by *Cla*I (expected size: 7442 bp), lanes 3, 6, 9: undigested gDNA (expected size: 9381 bp).

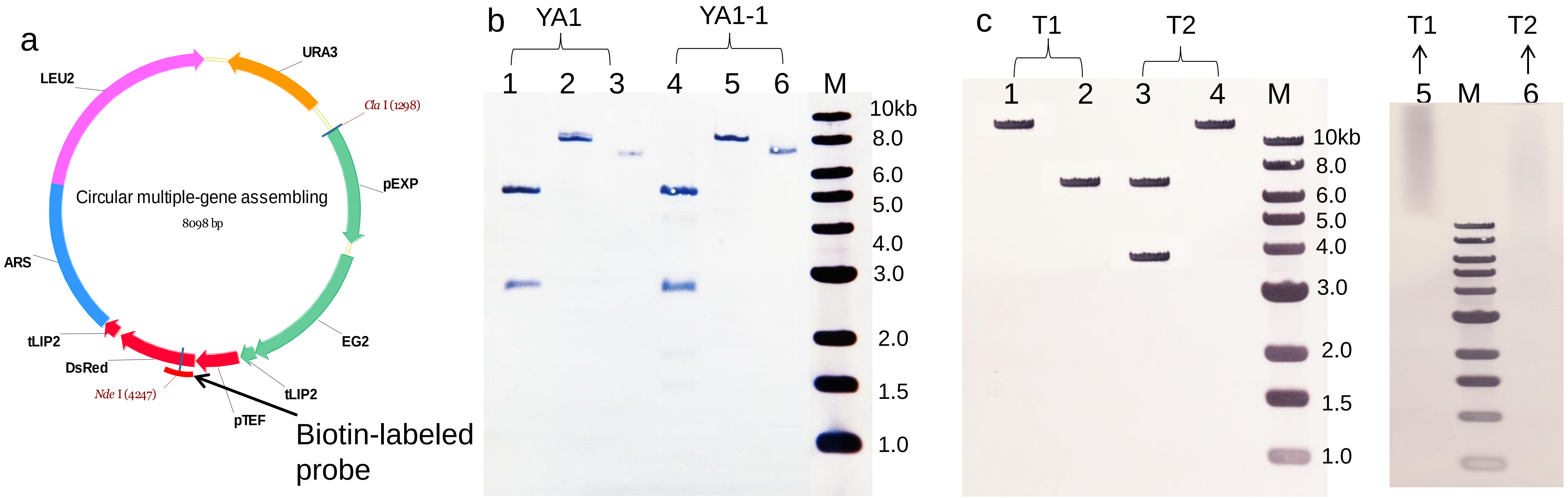

Supplementary Figure 4 Verification by Southern blot and restriction enzyme analysis of gene assembling of DNA fragments containing ARS but not telomeric sequences: formation of a circular plasmid. (a) Configuration of the obtained DNA assembly with the indications of the restriction enzyme sites and the position of the DNA probe; (b) Southern blot analysis of the DNA assemblies in wild type strain, initial transformants (YA1) and *Y. lipolytica* transformed with the linear DNA fragment isolated from YA1 (YA1-1) using the specific DNA probe, lanes 1 and 4: genomic DNA digested by *Cla*I/*Nde*I, expected bands: 2.9kb/5.1kb; lanes 2 and 5: genomic DNA digested by *Cla*I, expected fragments: 8.1kb; lanes 3 and 6: undigested genomic DNA; (c) Southern blot analysis of the DNA assemblies randomly incorporated into the genome of two different transformants (T1 and T2) using the specific DNA probe; lanes 1 and 3: genomic DNA digested by *Cla*I/*Nde*I; lanes 2 and 4: genomic DNA digested by *Cla*I; lanes 5 and 6: undigested genomic DNA.

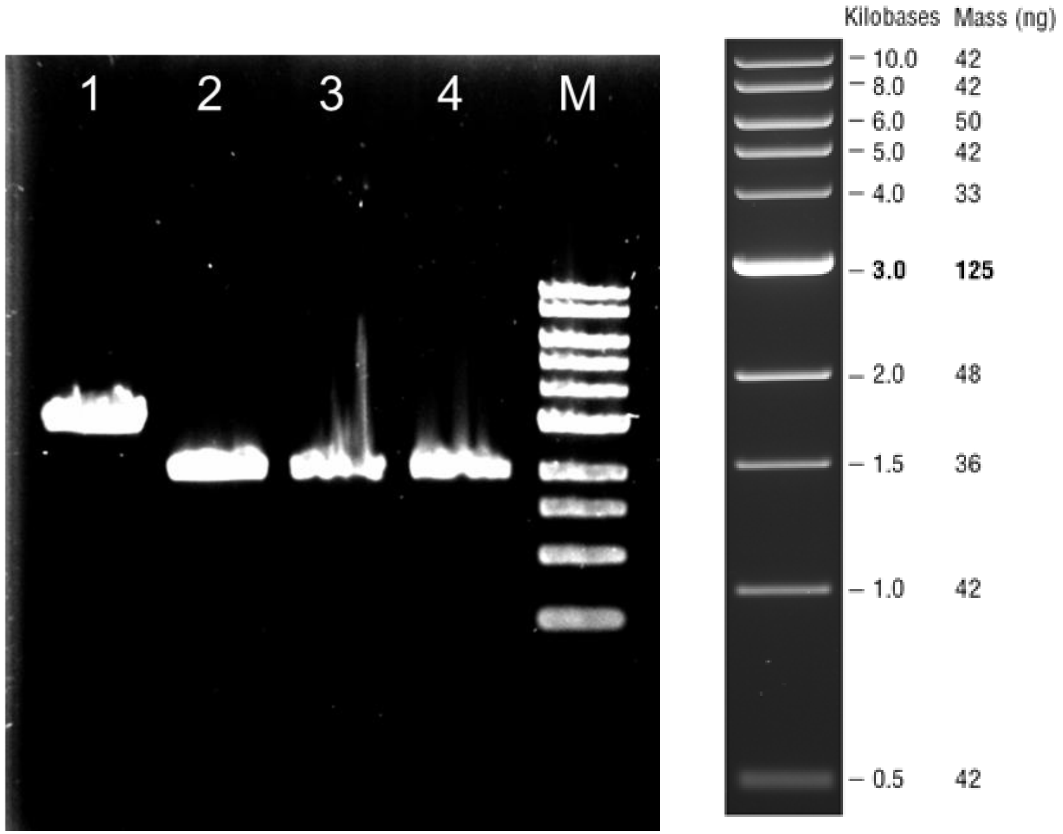

Supplementary Figure 5 PCR verification of the deletion of *HEM1* gene using primers ylHEM1F/ylHEM1R. lane 1, Po1d control, expected size: 2938bp; lanes 2 to 4, three independently colonies of Po1d transformants selected from YNB plate supplemented with 5-ALA, expected size: 2035bp.

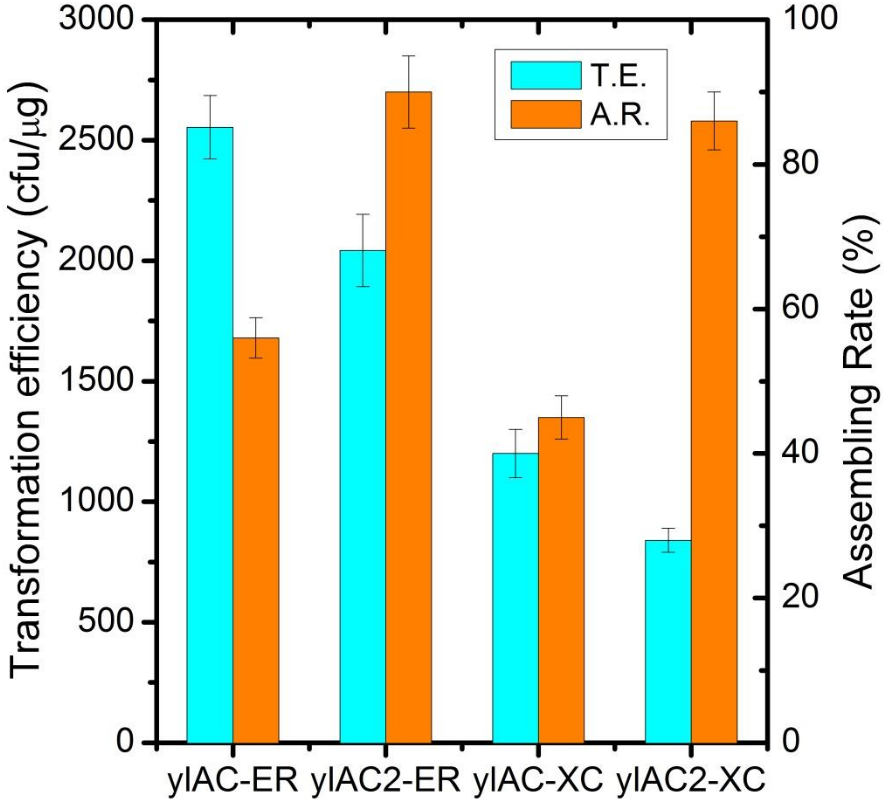

Supplementary Figure 6 Comparison of the transformation efficiency and assembling rate of ylAC2 with ylAC in multiple gene assembling. Shown are: ylAC-ER and ylAC-XC, ylAC for the assembling of *EG2* and *RedStar2*, Xylose and cellobiose co-consumption pathways, respectively; ylAC2-ER and ylAC2-XC, ylAC2 for the assembling of *EG2* and *RedStar2*, Xylose and cellobiose co-consumption pathways, respectively. The results were calculated from at least three biological replicates, error bars are standard deviation.

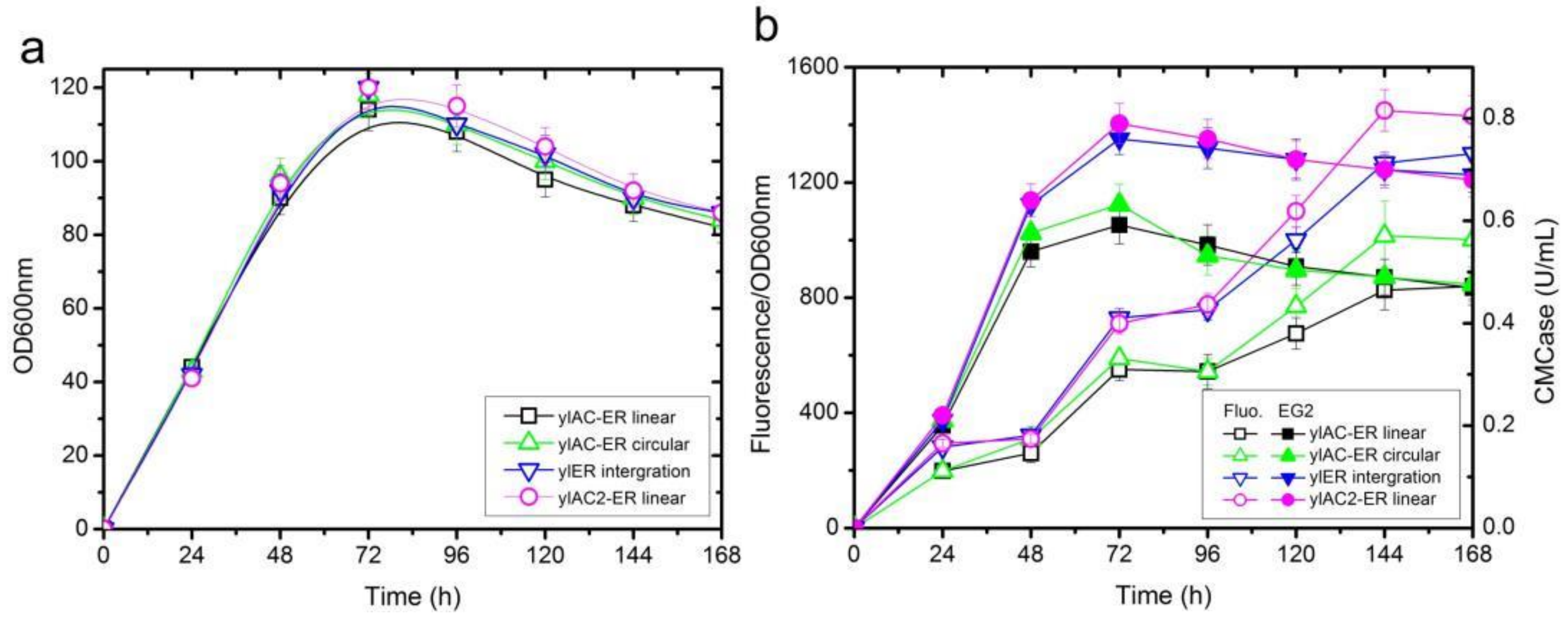

Supplementary Figure 7 Comparison of the growth and production of both EG2 and DsRed using artificial chromosome ylAC and ylAC2 system (linear DNA assembly ylAC-ER and ylAC2-ER, and circular DNA assembly ylAC-ER) with genome integration in *Y. lipolytica* (ylER). (a) Growth (b) Intensity of fluorescence and endoglucanase activity in the supernatant of the culture. The results were calculated from at least three biological replicates, error bars are standard deviation.

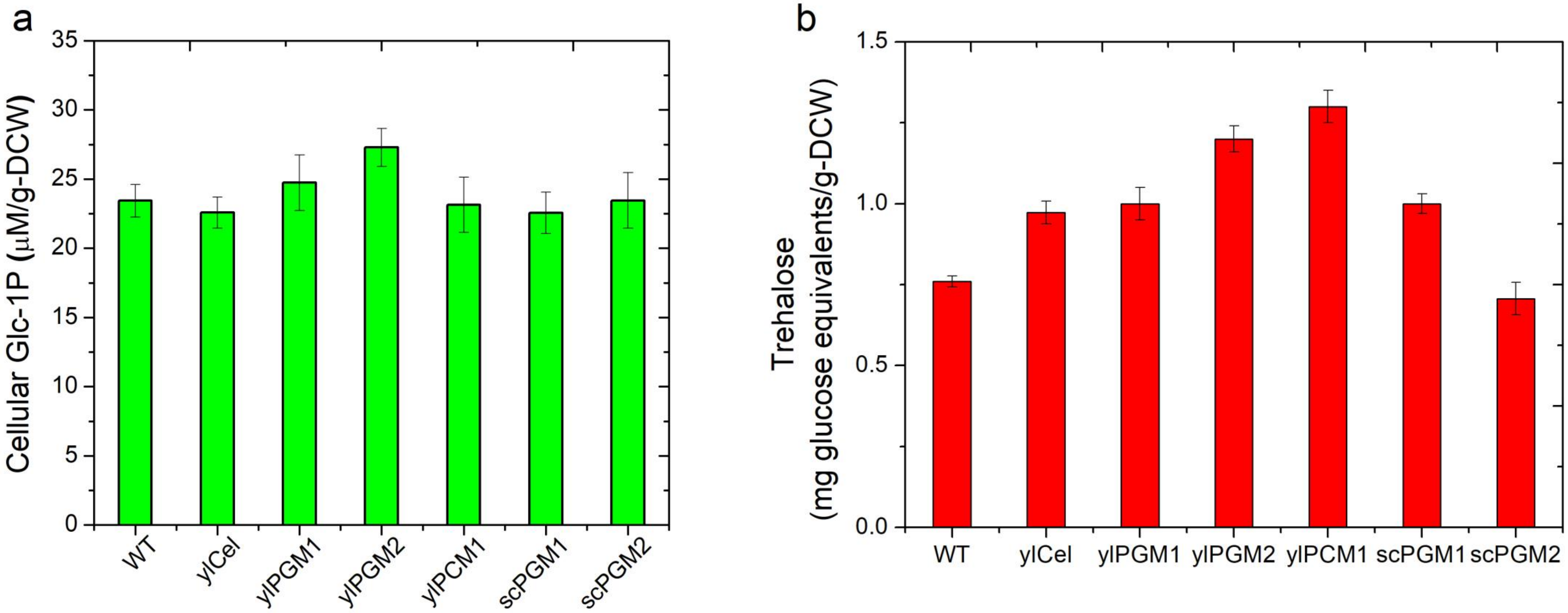

Supplementary Figure 8 Application of ylAC for the optimization of cellobiose phosphorolysis pathway in the yeast *Y. lipolytica*. (a) Cellular content of G1P; (b) Reserve carbon source trehalose in phosphorolytic *Y. lipolytica* (ylCello, and ylCello expressing different PGMs) compared with WT strain in aerobic growth on 10 g/L cellobiose. ylCBP expressing only cellobiose phosphorylase (CBP) was unable to grow on cellobiose as the sole carbon source, despite the fact that phosphorylase activity was detectable (0.45±0.02 U/mg total protein). The representative data of the samples taken from the exponential growth phase are shown. Results were calculated from at least five biological replicates and are given as the mean value and standard deviation.

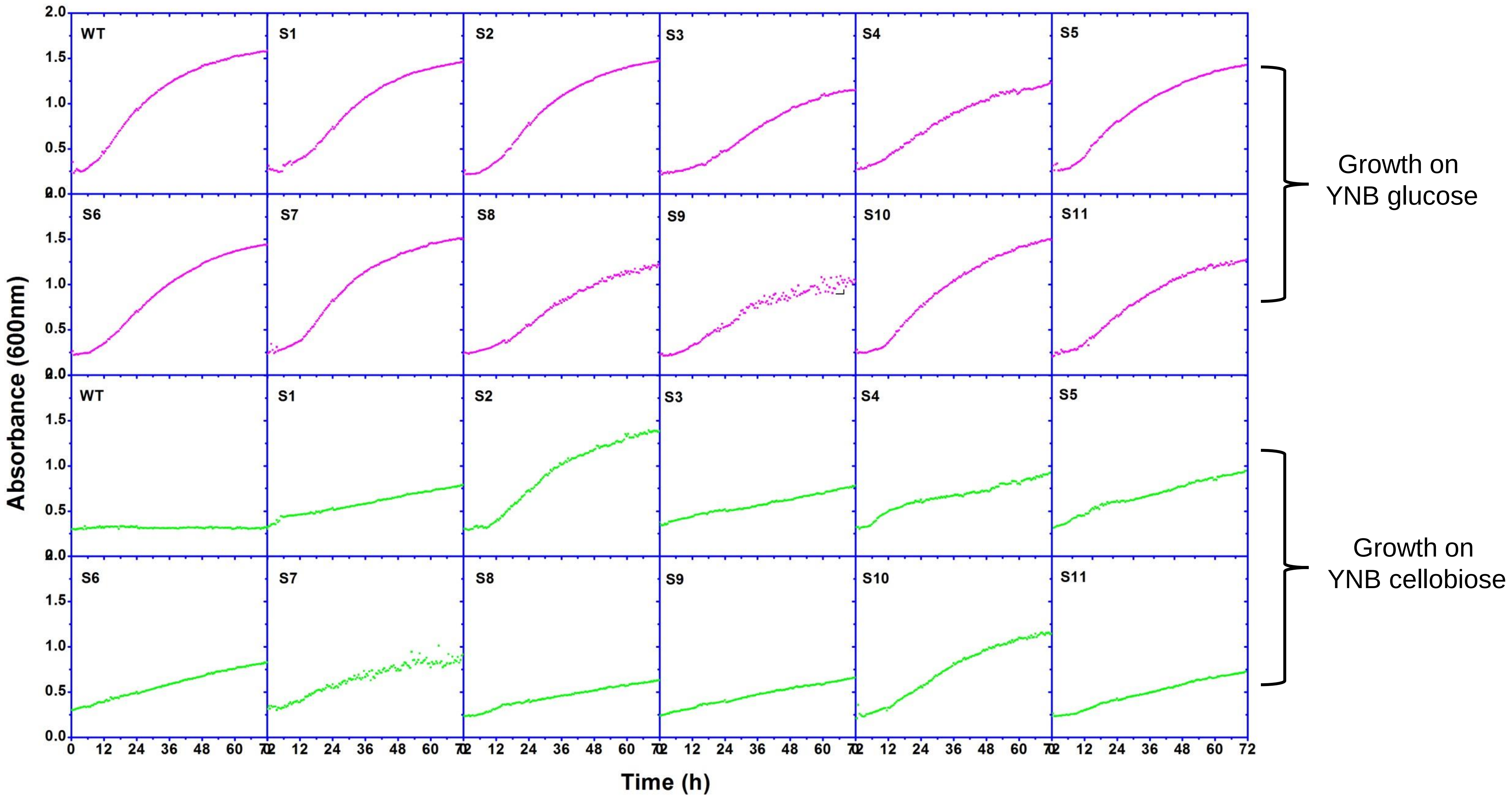

Supplementary Figure 9 Comparison of *Y. lipolytica* expressing different PGMs in a ylAC-*CBP1-CDT1-scPGM2* assembly during aerobic growth on 5 g/L glucose or 5 g/L cellobiose as carbon source in 48-well plate. Figure shows absorbance at 600nm versus time. Each data point represents the mean of at least three independent experiments and the standard deviation is less than 5%.

Gene	Host	GenBank ACC. Number	Primers for verification	Target length (bp)	Figure
PGM1	*Y. lipolytica*	YALI0E02244g	*VGF1*/VGR1	1868	a
PGM2	*Y. lipolytica*	YALI0E02090g	*VGF1*/VGR2	1736	b
PCM1	*Y. lipolytica*	YALI0E29579g	*VGF1*/VGR3	1493	c
PGM1	*S. cerevisiae*	NM_001179693.1	*VGF1*/VGR4	1532	d
PGM2	*S. cerevisiae*	NM_001182605.1	*VGF1*/VGR5	1674	e

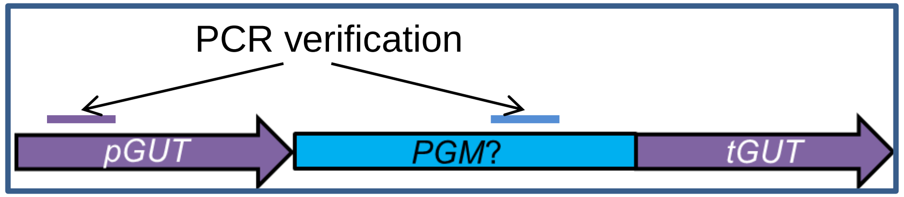

Total transformants verified: No. 1 to 30

a

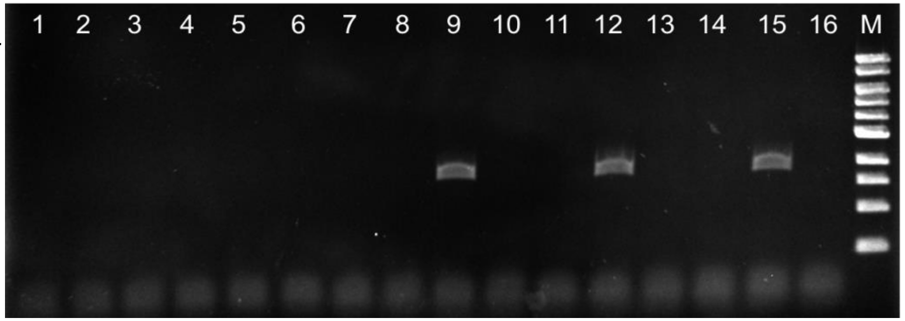

b

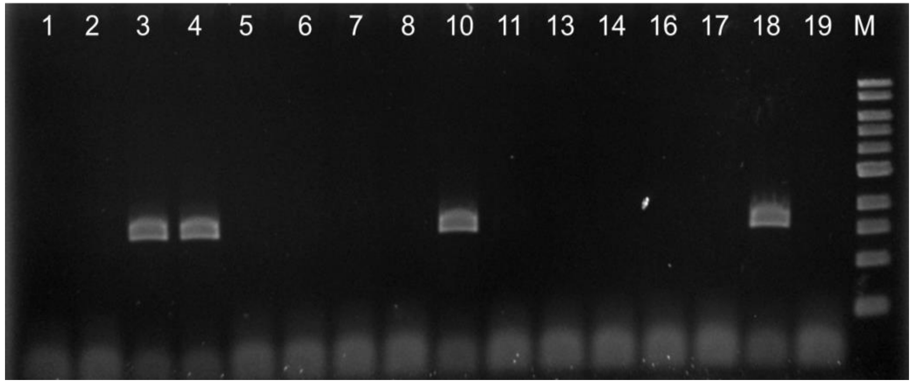

c

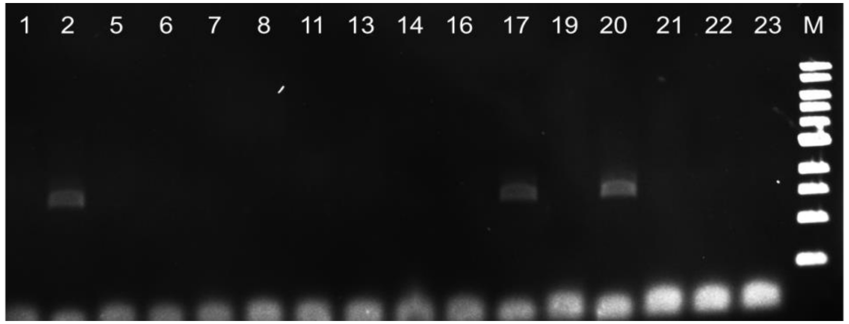

d

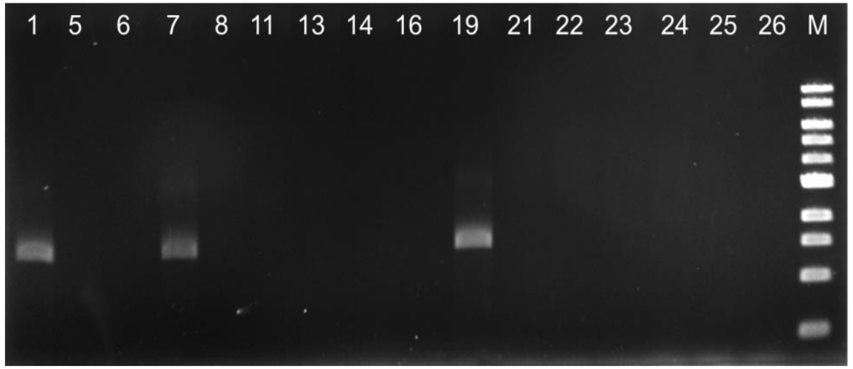

e

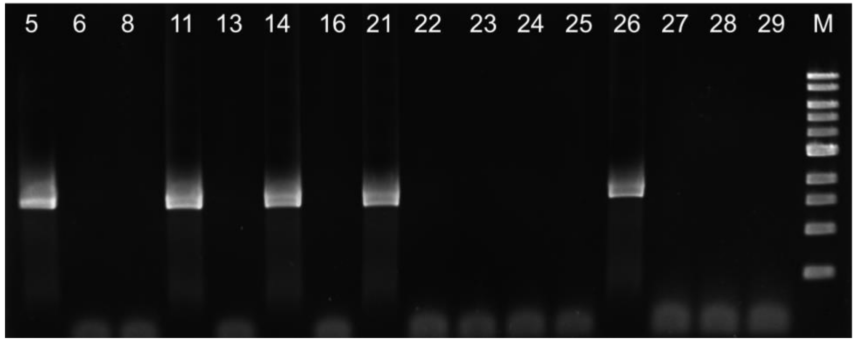

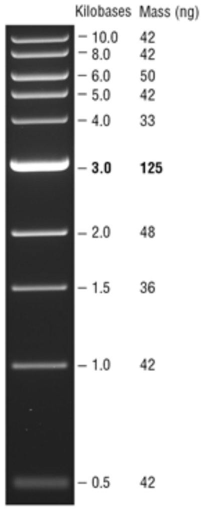

Supplementary Figure 10 PCR verification of PGM gene of expression.

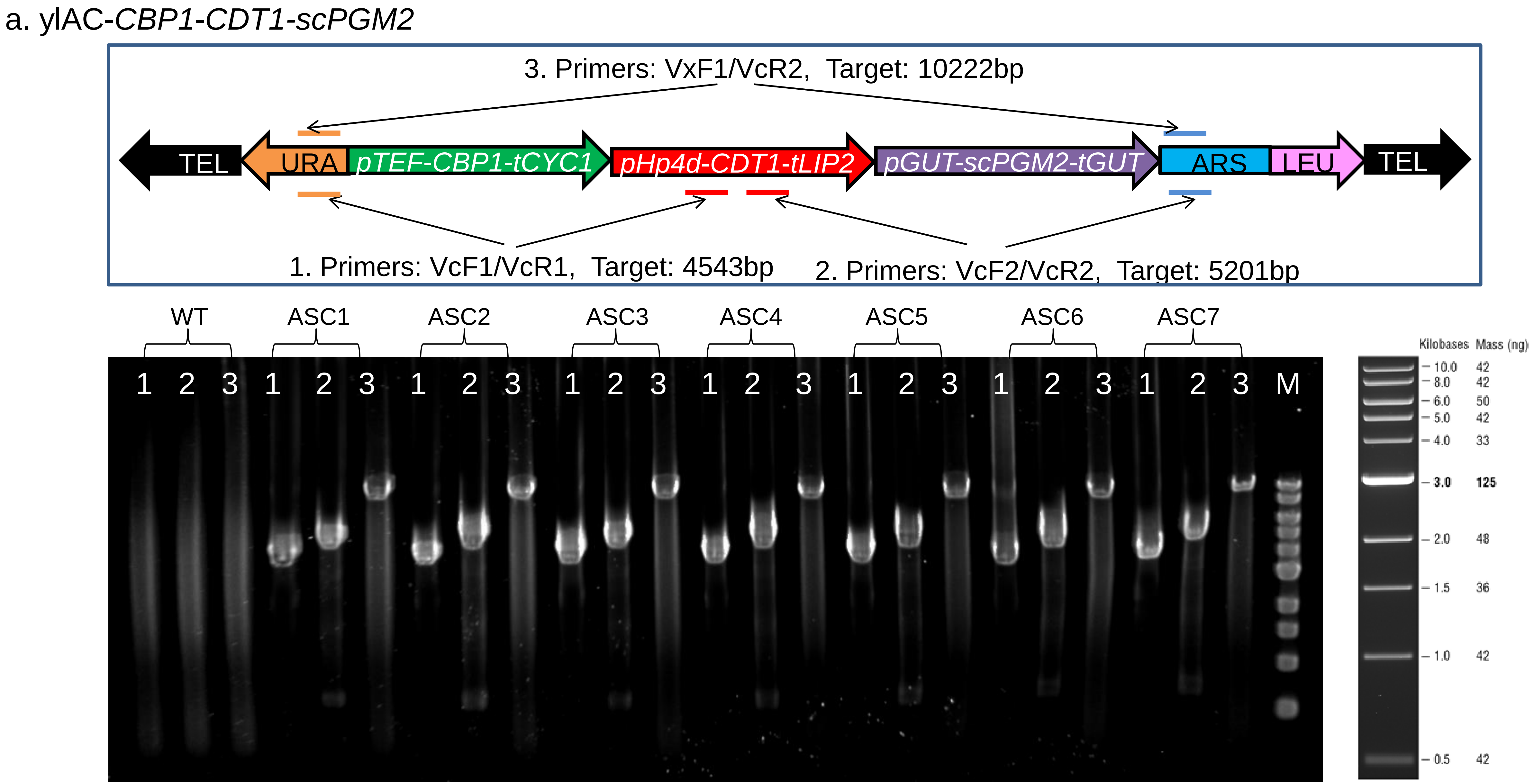

Supplementary Figure 11 PCR verification of assembling of cellobiose phosphorolysis pathway.

[a]60 transformants tested : 90% of correct assembly

b. *URA3-CBP1-CDT1-scPGM2-ARS-LEU2*

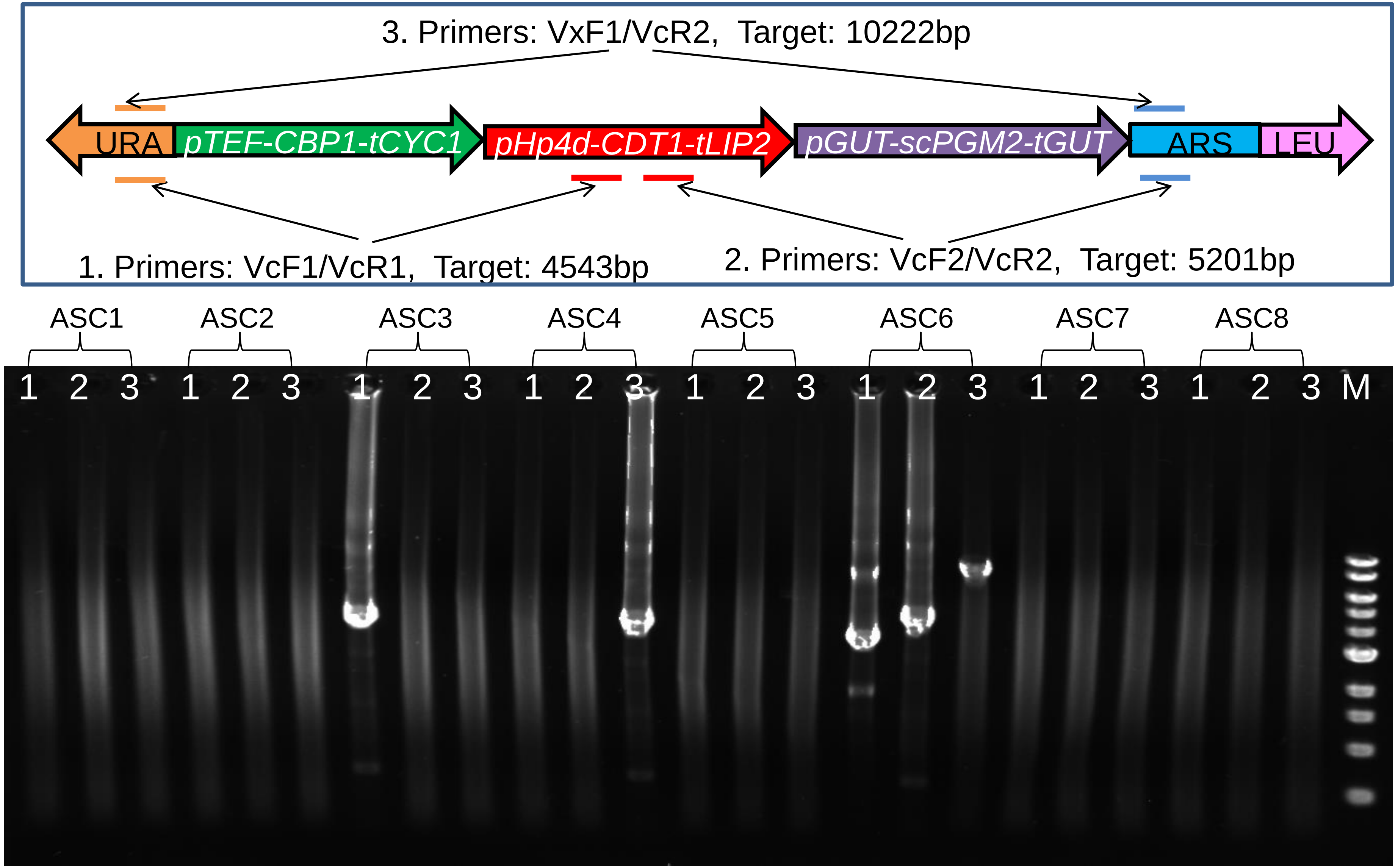

Supplementary Figure 11 PCR verification of assembling of cellobiose phosphorolysis pathway.

[b]60 transformants tested: 20% of correct assembly

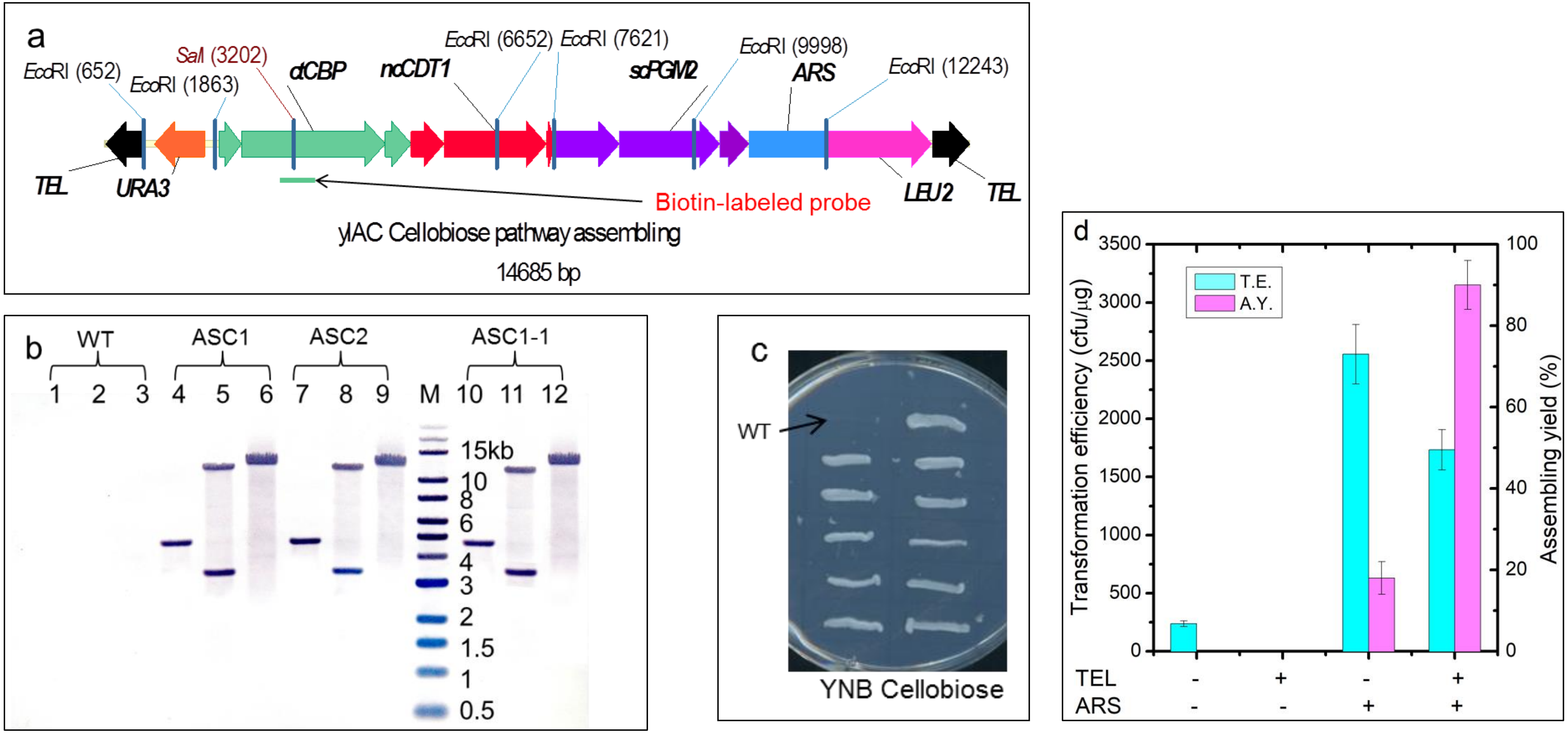

Supplementary Figure 12 Verification of the functionality of the DNA assembly of cellobiose phosphorolysis pathway as extrachromosomal replicating linear DNA. (a) Configuration of the desired DNA assembly with the indications of the restriction enzyme sites and position of the DNA probe; (b) Southern blot analysis of the DNA assemblies in wild type strain, initial transformants (ASC1 and 2), and *Y. lipolytica* transformed with the linear DNA fragment isolated from ASC1 (ASC1-1) using the specific DNA probe, lanes 1, 4 and 7: genomic DNA digested by *Eco*RI (expected bands: 4.8kb), lanes 2, 5 and 8: genomic DNA digested by *Sal*I (expected bands: 3.2/11.5kb), lanes 3, 6 and 9: undigested genomic DNA (expected bands: 14.7kb); (c) Activation of cellobiose consumption in ACS1-1 transformants; (d) the transformation efficiency and assembling yield of DNA assembling.

- ylAC-*XYL1-XYL2-XKS1-CBP-CDT-scPGM2*

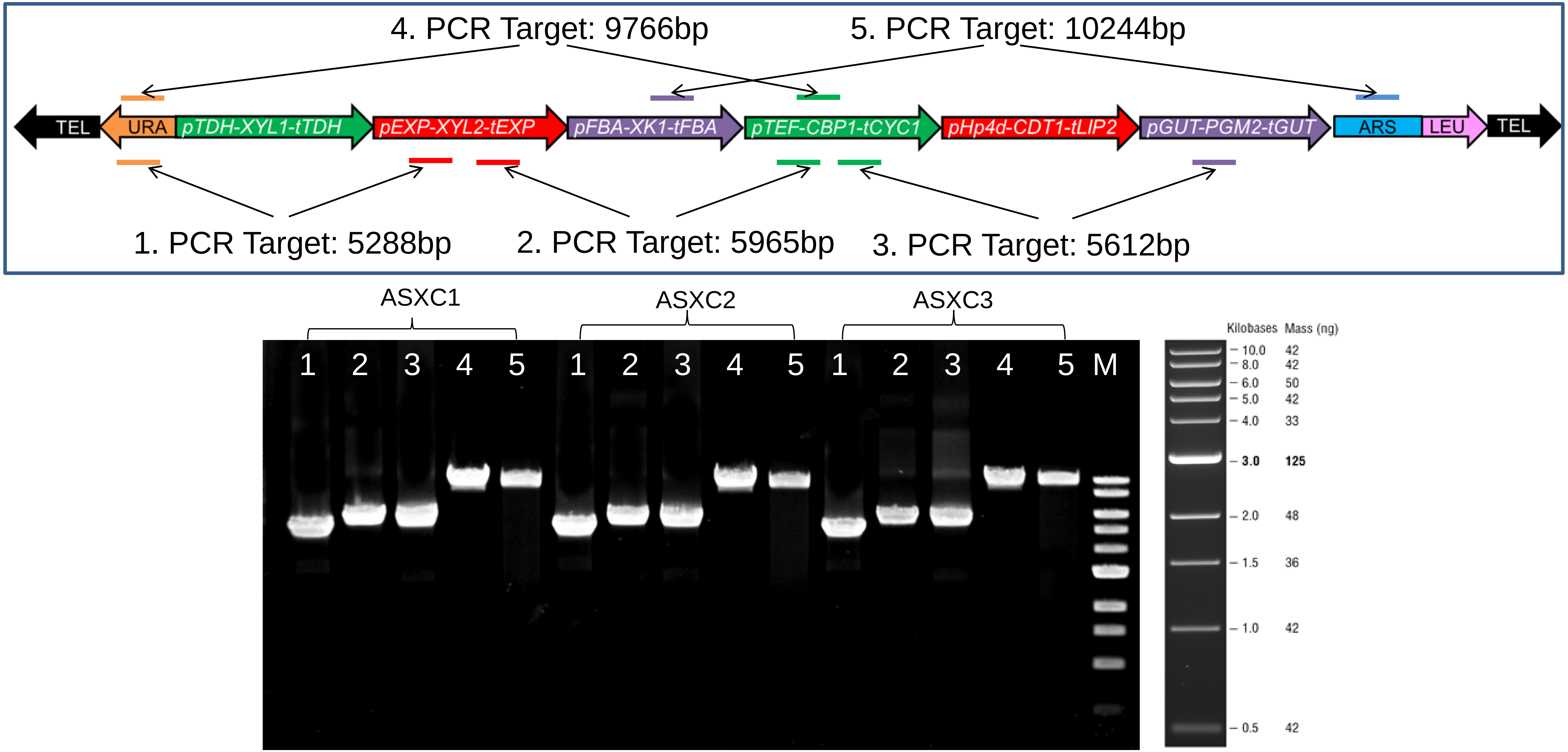

Supplementary Figure 13 PCR verification of assembling of cellobiose phosphorolysis and xylose consumption pathways.

60 transformants tested: 70% of correct assembly

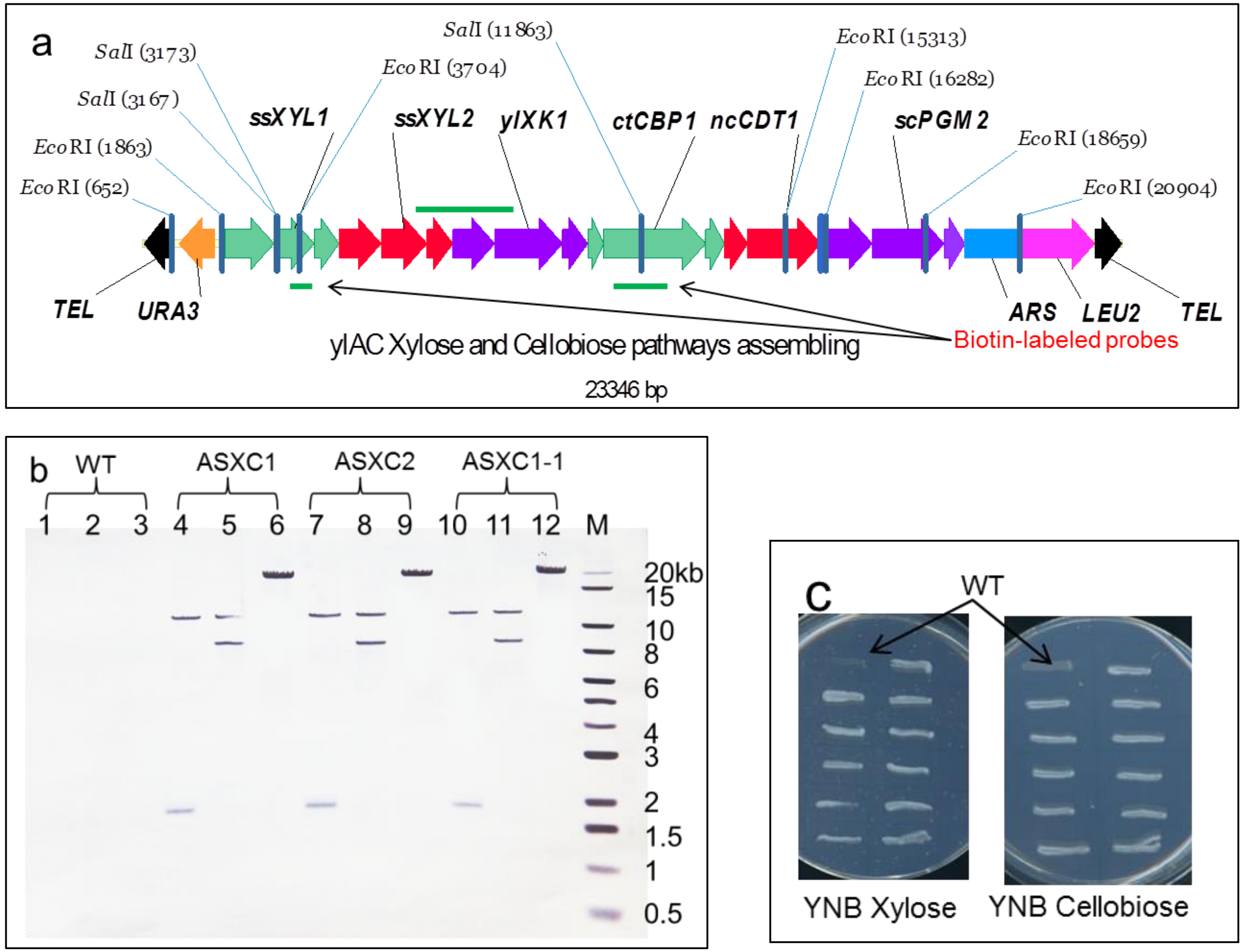

Supplementary Figure 14 Verification of the functionality of the DNA assembly of cellobiose phosphorolysis pathway and xylose consumption pathway as extrachromosomal replicating linear DNA. (a) Configuration of the desired DNA assembly with the indications of the restriction enzyme sites and positions of the DNA probes; (b) Southern blot analysis of the DNA assemblies in wild type strain, initial transformants (ASXC1 and 2), and *Y. lipolytica* transformed with the linear DNA fragment isolated from ASXC1 (ASXC1-1) using the specific DNA probes, lanes 1, 4 and 7: genomic DNA digested by *Eco*RI (expected bands: 1.8/11.6kb), lanes 2, 5 and 8: genomic DNA digested by *Sal*I (expected bands: 8.7/11.5kb), lanes 3, 6 and 9: undigested genomic DNA (expected bands: 23.3kb); (c) Activation of cellobiose and xylose consumption of transformants ASXC1-1.

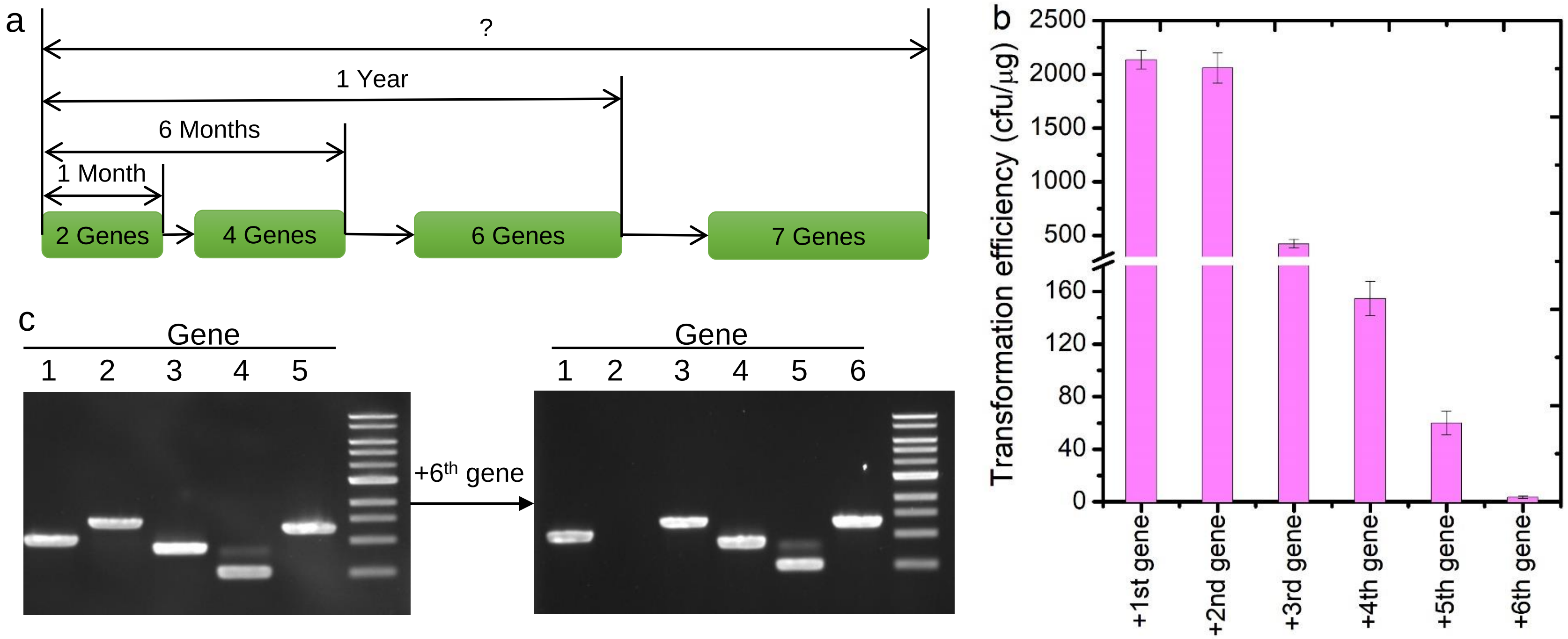

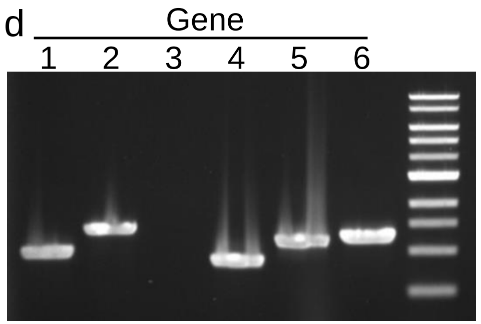

Supplementary Figure 15 Challenges in multi-gene integration and expression. **a** Time line for the integration of 7 genes. **b** Decrease in the transformation efficiency along with the number of genes being integrated into the genome. **c** gene replacement event: PCR verification of the presence of the genes in the genome of *Y. lipolytica* showed the introducing the following gene (6th) can knock out the gene (2nd) that has been introduced previously. **d** gene loss (the 3rd gene) during the excision of the selection marker using *Cre/Loxp*.

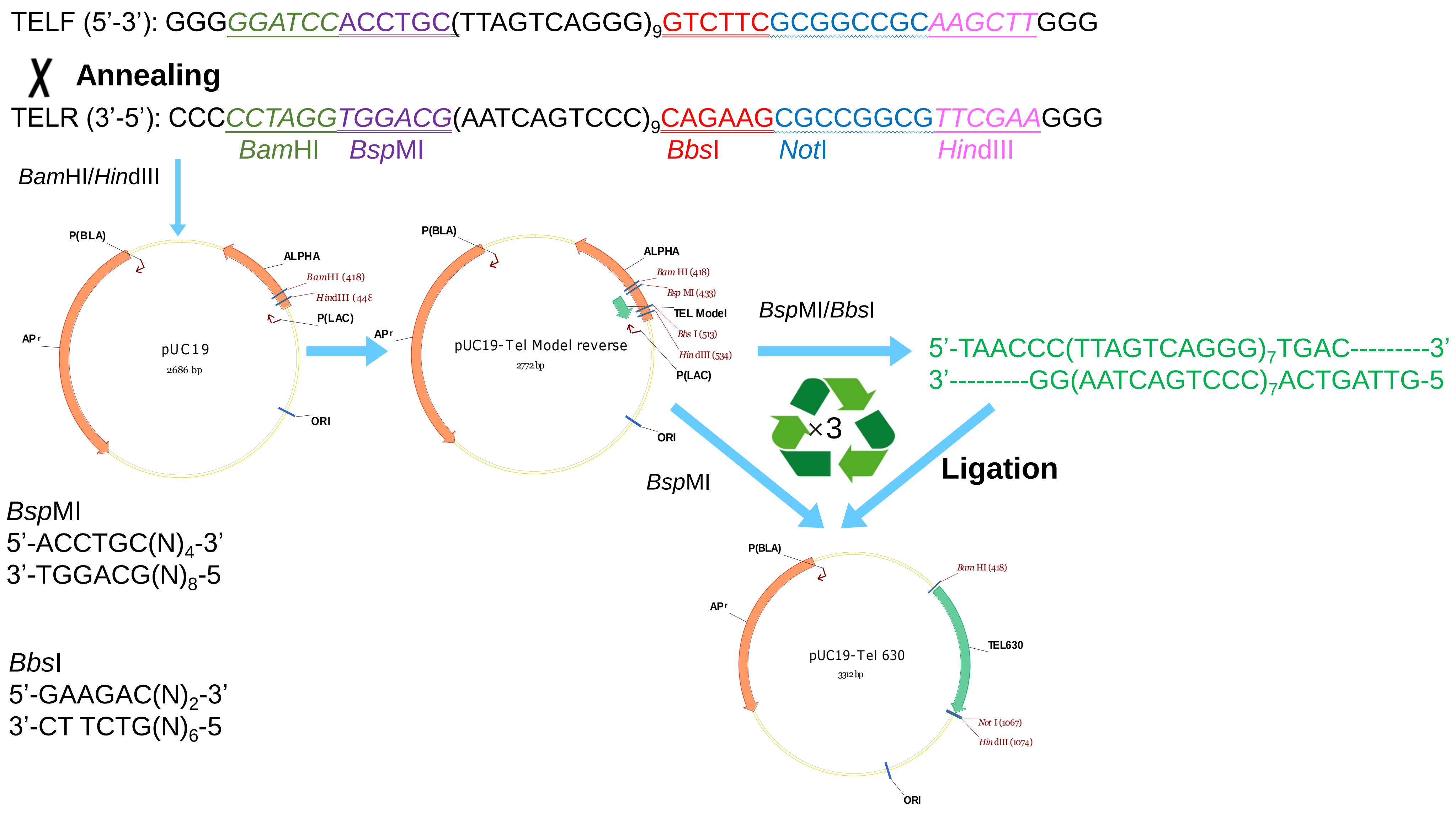

Supplementary Figure 16 Strategy for synthesis of model telomere.

*Telomeric repeats: 5’-TTAGTCAGGG-3’ decameres tandemly repeated 40–50 times

- **Ligation of pUC19 with annealed TEL(9)* oligonucleotides**

Agarose gel (2%)

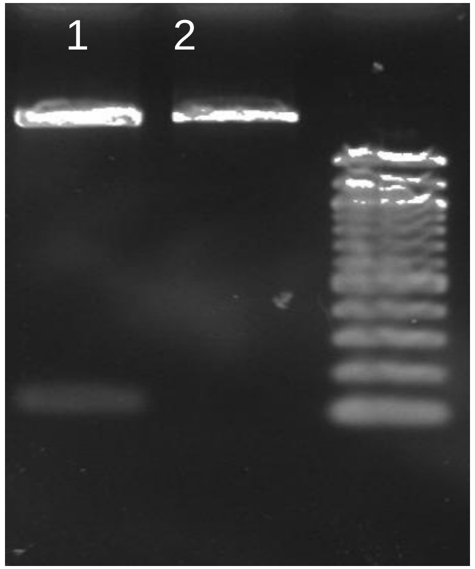

Lane 1, pUC19-TEL**(9)** digested by *Bam*HI/*Hin*dIII; Lane 2, pUC19 digested by *Bam*HI/*Hin*dIII.

- **Ligation of pUC19-TEL(9) with purified TEL(9) DNA.**

Agarose gel (2%)

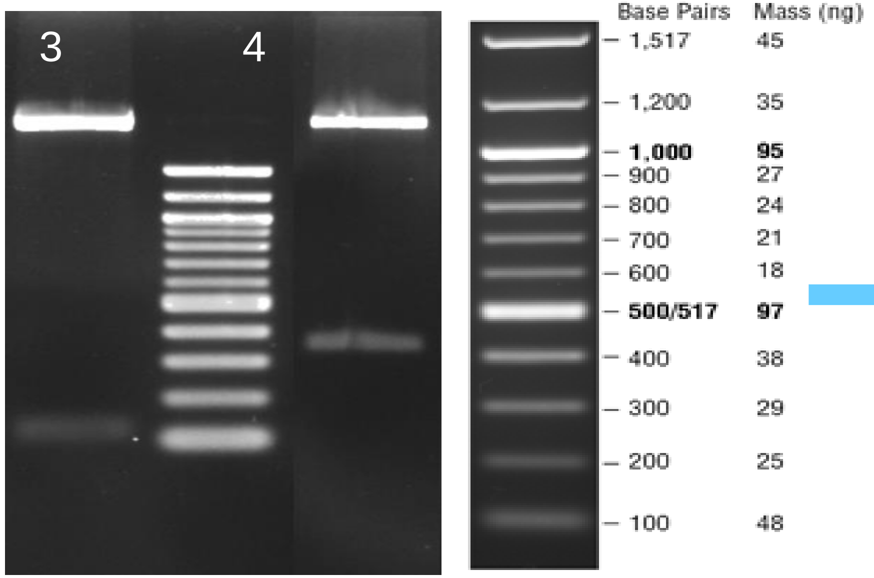

Lane 3, 4, pUC19-TEL(9) and pUC19-TEL**(39)** digested by *Bam*HI/*Hin*dIII, respectively.

- **Ligation of pUC19-TEL(39) with purified TEL(9) DNA.**

Agarose gel (1.3%)

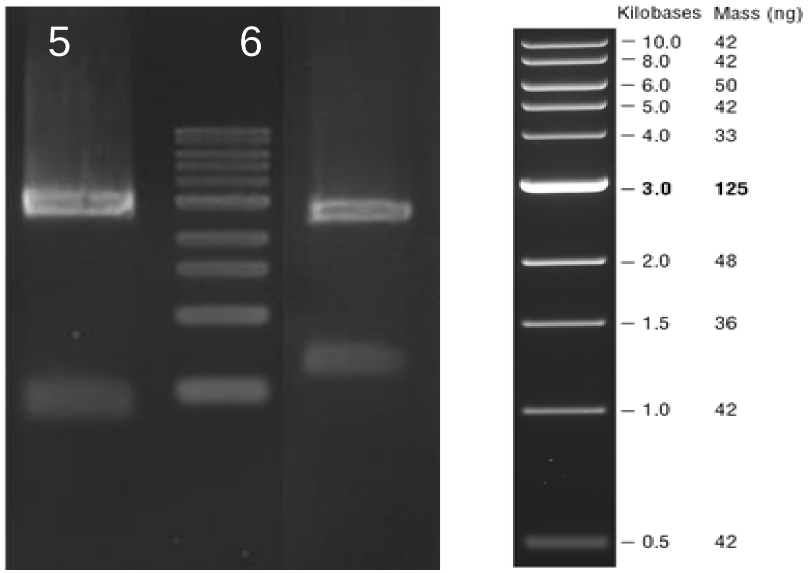

Lane 5, 6, pUC19-TEL(39) and pUC19-TEL**(63)** digested by *Bam*HI/*Hin*dIII, respectively.

Supplementary Figure 17 Verification of a synthesized model telomere by DNA electrophoresis.

*TEL(n)=TEL(the number of telomeric repeats)

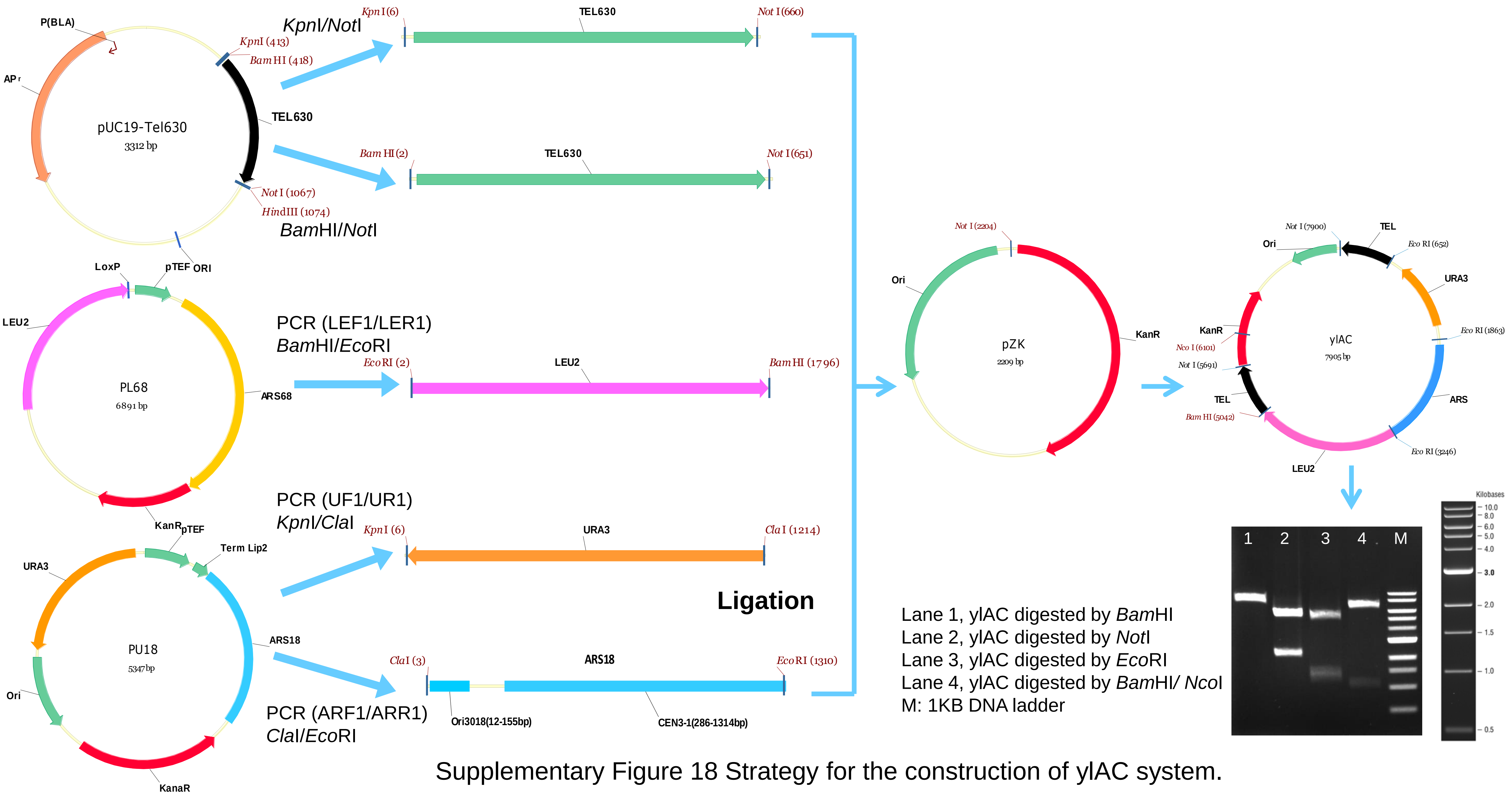

Supplementary Figure 18 Strategy for the construction of ylAC system.

Supplementary Tables

Supplementary Table 1. The promoters and terminators characterized in the present study

Proteins of target	Gene locus tag	Promoter (bp)	Terminator (bp)
EXP (Export protein)[1]	YALI0C12034g	-999 to -1	623
FBA (Fructose-bisphosphate aldolase)[1]	YALI0E26004g	-996 to -1	599
GPAT (Glycerol-3-phosphate o-acyltransferase)[1]	YALI0C00209g	-1122 to -1	587
GPD (Glycerol-3-phosphate dehydrogenase)	YALI0C00209g	-1184 to -1	580
GUT (Glycerol-3-phosphate dehydrogenase mitochondrial)	YALI0_B13970g	-1102 to -1	490
HXK1 (Hexokinase I)	YALI0B22308g	-1026 to -1	576
HXK2 (Hexokinase II)	YALI0E20207g	-1040 to -1	431
HXK3 (Hexokinase 3)	YALI0E15488g	-999 to -1	506
PGK (Phosphoglycerate kinase)	YALI0D12400g	-987 to -1	481
TDH (Glyceraldehyde-3-phosphate dehydrogenase)[1]	YALI0C06369g	-927 to -1	599
TPI (Triose-phosphate isomerase)	YALI0F05214g	-1112 to -1	522
TEF (Translation elongation factor-1)	YALI0C09141g	-366 to -41	LIP2 (127)

Supplementary Table 2 Determination of the copy number of linear and circular DNA assemblies

Sample	Gene	Cq	Copy number of the molecules
Linear DNA assemblies			
1	Actin	18.27±0.09	13 944±774
	RedStar2	18.4±0.01	14 054±91
2	Actin	18.33±0.10	13 423±823
	RedStar2	17.54±0.04	24 662±636
3	Actin	18.36±0.02	13 170±166
	RedStar2	17.33±0.06	28 293±1088
4	Actin	17.65±0.03	20 666±389
	RedStar2	17.43±0.02	26 502±344
5	Actin	17.8±0.04	18 790±470
	RedStar2	17.69±0.02	22 358±290
6	Actin	17.67±0.02	20 406±257
	RedStar2	17.68±0.01	22 505±146
Circular DNA assemblies			
1	Actin	17.79±0.05	18 909±590
	RedStar2	16.67±0.09	43 563±2489
2	Actin	18.1±0.08	15 532±768
	RedStar2	16.67±0.04	43 563±1124
3	Actin	17.9±0.07	17 634±766
	RedStar2	16.66±0.12	43 849±3309
4	Actin	17.71±0.12	19 894±1458
	RedStar2	17.54±0.03	24 663±479
5	Actin	17.88±0.03	17 860±336
	RedStar2	17.26±0.02	29 618±384
6	Actin	17.72±0.01	19 768±125
	RedStar2	16.94±0.07	36 512±1633

Results were calculated from at least six biological replicates and are given as the mean value ± standard deviation.

Supplementary Table 3 Comparison of growth and biomass yield of *Y. lipolytica* Po1d control (WT) and recombinant strains in aerobic glucose and cellobiose cultivation

Parameter	$\mu_{max\text{-}glc}$ (h^{-1})	$\mu_{max\text{-}cello}$ (h^{-1})	$Y_{X/S}$ (DCW-g/g-glc)	$Y_{X/S}$ (DCW-g/g-cello)	Residue cello 60h (%)
WT	0.16±0.01	N.A.	0.54±0.01	N.A.	N.A.
ylCBP	0.16±0.02	N.A.	0.55±0.01	N.A.	N.A.
ylCello	0.15±0.01	0.08±0.01	0.52±0.03	0.46±0.00	18.0±1.0
ylCello-ylPGM1	0.15±0.01	0.08±0.00	0.53±0.02	0.47±0.01	17.5±0.5
ylCello-ylPGM2	0.16±0.00	0.06±0.00	0.54±0.00	0.43±0.02	19.6±1.2
ylCello-ylPCM1	0.15±0.01	0.09±0.01	0.53±0.01	0.45±0.02	17.5±0.9
ylCello-scPGM1	0.16±0.00	0.10±0.00	0.54±0.02	0.48±0.01	13.6±0.8
ylCello-scPGM2	0.16±0.01	0.15±0.01	0.53±0.02	0.52±0.01	0

Results were calculated from at least three biological replicates and are given as the mean value ± standard deviation. N.A.= Not available

Growth was only possible when the cellodextrin transporter CDT1 was also present in the cell (ylCello). However, the growth rate and biomass yield of ylCello were only 50% and 85%, respectively, of those of the WT grown on glucose.

Supplementary Table 4 Microbial strains used in the present study

Strains	Relevant genotype	Source of reference
E. coli DH5	Φ80dlacZΔm15, *recA1*, *endA1*, *gyrA96*, *thi-1*, *hsdR17* (rk^-, mk^+), *supE44*, *relA1*, *deoR*, Δ(*lacZY*A-argF) U169	Invitrogen
Y. lipolytica Po1d	*MATA*, *ura3-302*, *leu2-270*	[2]
Y. lipolytica JMY1212 Zeta	*MATA, ura3-302, leu2-270-LEU2-zeta, xpr2-322*	[3]
Zeta-ptEXP	Zeta, *pEXP-RedStar2-tEXP*	This investigation
Zeta-ptFBA	Zeta, *pFBA-RedStar2-tFBA*	This investigation
Zeta-ptGPAT	Zeta, *pGPAT-RedStar2-tGPAT*	This investigation
Zeta-ptGPD	Zeta, *pGPD-RedStar2-tGPD*	This investigation
Zeta-ptGUT	Zeta, *pGUT-RedStar2-tGUT*	This investigation
Zeta-ptHXK1	Zeta, *pHXK1-RedStar2-tHXK1*	This investigation
Zeta-ptHXK2	Zeta, *pHXK2-RedStar2-tHXK2*	This investigation
Zeta-ptHXK3	Zeta, *pHXK3-RedStar2-tHXK3*	This investigation
Zeta-ptPGK	Zeta, *pPGK-RedStar2-tPGK*	This investigation
Zeta-ptTDH	Zeta, *pTDH-RedStar2-tTDH*	This investigation
Zeta-ptTPI	Zeta, *pTPI-RedStar2-tTPI*	This investigation
Zeta-pTEF/tLIP2	Zeta, *pTEF-RedStar2-tEXP*	This investigation
WT	*MATA*, *ura3-302-URA3*, *leu2-270*-LEU2	This investigation
ylCel	*pTEF-cbp1, php4d-CDT1*	This investigation
ylPGM1	*pTEF-cbp1, php4d-CDT1, pGUT-ylPGM1*	This investigation
ylPGM2	*pTEF-cbp1, php4d-CDT1, pGUT-ylPGM2*	This investigation
ylPCM1	*pTEF-cbp1, php4d-CDT1, pGUT-ylPCM1*	This investigation
scPGM1	*pTEF-cbp1, php4d-CDT1, pGUT-ylPGM1*	This investigation
scPGM2	*pTEF-cbp1, php4d-CDT1, pGUT-ylPGM2*	This investigation
YTA1	ylAC-*EG2-RedStar2* assembly (linear)	This investigation
YT1	*URA-EG2-RedStar2-ARS-LEU2* assembly (circular)	This investigation
ASC1	ylAC-*CBP1-CDT1-scPGM2* assembly	This investigation
ASXC1	ylAC-*XYL1-XYL2-XKS1-CBP-CDT-scPGM2* assembly	This investigation
Y. lipolytica Po1dh	*MATA*, *ura3-302*, *leu2-270*, *hem1-903*	This investigation
ylAC2-ER	Po1dh, ylAC2-*EG2-RedStar2* assembly	This investigation
ylAC2-XC	Po1dh, ylAC2-*XYL1-XYL2-XKS1-CBP-CDT-scPGM2* assembly	This investigation

Supplementary Table 5. Plasmids used or constructed in the present study

Plasmids	Description	Source of reference
pUC19		Invitrogen
pUC19-Tel Model	telomeric repeats (n=9)	This investigation
pUC19-Tel630	telomeric repeats (n=63)	This investigation
JMP62UraTEF	*URA3*, *pTEF*, *tLIP2*	[2]
JMP62LeuTEF	*LEU2*, *pTEF*, *tLIP2*	[2]
JMP62LEU2expTEF-RedStar2	*LEU2*, *pTEF-RedStar2- tLIP2*	[4]
JMP62Urahp4d	*URA3*, *php4d*, *tLIP2*	[5]
pU18	*ARS18, URA3*	[6]
pZK		This investigation
ylAC	*URA3*, *LEU2*, *ARS*, *TEL*	This investigation
YlAC2	*URA3*, *LEU2*, *HEM1*, *ARS*, *TEL*	This investigation
pZP-EXP	*URA3*, *pEXP*, *tEXP*	This investigation
pZP-FBA	*URA3*, *pFBA*, *tFBA*	This investigation
pZP-GPAT	*URA3*, *pGPAT*, *tGPAT*	This investigation
pZP-GPD	*URA3*, *pGPD*, *tGPD*	This investigation
pZP-GUT	*URA3*, *pGUT*, *tGUT*	This investigation
pZP-HXK1	*URA3*, *pHXK1*, *tHXK1*	This investigation
pZP-HXK2	*URA3*, *pHXK2*, *tHXK2*	This investigation
pZP-HXK3	*URA3*, *pHXK3*, *tHXK3*	This investigation
pZP-PGK	*URA3*, *pPGK*, *tPGK*	This investigation
pZP-TDH	*URA3*, *pTDH*, *tTDH*	This investigation
pZP-TPI	*URA3*, *pTPI*, *tTPI*	This investigation
pZP-EXP-DsRed	*URA3*, *pEXP-RedStar2-tEXP*	This investigation
pZP-FBA-DsRed	*URA3*, *pFBA-RedStar2-tFBA*	This investigation
pZP-GPAT-DsRed	*URA3*, *pGPAT-RedStar2-tGPAT*	This investigation
pZP-GPD-DsRed	*URA3*, *pGPD-RedStar2-tGPD*	This investigation
pZP-GUT-DsRed	*URA3*, *pGUT-RedStar2-tGUT*	This investigation
pZP-HXK1-DsRed	*URA3*, *pHXK1-RedStar2-tHXK1*	This investigation
pZP-HXK2-DsRed	*URA3*, *pHXK2-RedStar2-tHXK2*	This investigation
pZP-HXK3-DsRed	*URA3*, *pHXK3-RedStar2-tHXK3*	This investigation
pZP-PGK-DsRed	*URA3*, *pPGK-RedStar2-tPGK*	This investigation
pZP-TDH-DsRed	*URA3*, *pTDH-RedStar2-tTDH*	This investigation
pZP-TPI-DsRed	*URA3*, *pTPI-RedStar2-tTPI*	This investigation
pZP-TDH-XR	*URA3*, *pTDH-ssXYL1-tTDH*	This investigation
pZP-EXP-XDH	*URA3*, *pEXP-ssXYL2-tEXP*	This investigation
pZP-FBA-XKS	*URA3*, *pFBA-ylXKS1-tFBA*	This investigation
JMP62UraTCbp1	*URA3*, *pTEF-CBP1-tLIP2*	This investigation
JMP62UrahCdt1	*URA3*, *php4d-CDT1-tLIP2*	This investigation
JMP62UraTCbp1tCYC	*URA3*, *pTEF-CBP1-tCYC1*	This investigation
pZP-GUT-ylPGM1	*URA3*, *pGUT-ylPGM1-tGUT*	This investigation
pZP-GUT-ylPGM2	*URA3*, *pGUT-ylPGM2-tGUT*	This investigation

pZP-GUT-ylPCM1	*URA3*, *pGUT-ylPCM1-tGUT*	This investigation
pZP-GUT-scPGM1	*URA3*, *pGUT-scPGM1-tGUT*	This investigation
pZP-GUT-scPGM2	*URA3*, *pGUT-scPGM2-tGUT*	This investigation
JMP62UraTB2EE2	*URA3*, *pHTEF-BGL2, pEXP-EG2*	[7]

Supplementary Methods

Plasmid construction and transformation.

(i) Construction and transformation of expression cassettes for DsRed under the control of various promoters and terminators. Endogenous gene sequences containing promoters, terminators and the open reading frame of the genes encoding export protein (EXP, primer pair ExpF1/ExpR1), fructose-bisphosphate aldolase (FBA1, FbaF1/FbaR1), glycerol-3-phosphate o-acyltransferase (GPAT, GpatF1/GpatR1), glycerol-3-phosphate dehydrogenase (GPD, GpdF1/GpdR1), mitochondrial glycerol-3-phosphate dehydrogenase (GUT, GutF1/GutR1), hexokinase I (HXK1, Hxk1F/Hxk1R), II (HXK2, Hxk2F/Hxk2R) and III (HXK3, Hxk3F/Hxk3F), phosphoglycerate kinase (PGK, PgkF1/PgkR1), glyceraldehyde-3-phosphate dehydrogenase (TDH, TdhF1/TdhR1) and triose-phosphate isomerase (TPI, TpiF1/TpiR1), were amplified from *Y. lipolytica* Po1d gDNA and ligated with the PCR fragment amplified from the plasmid JMP62UraTEF (primer pair ZPF/ZPR) using In-Fusion® HD Cloning Kit (Clontech, USA). Reporter gene *RedStar2* encoding fluorescence protein DsRed was amplified from the plasmid JMP62 LEU2ex pTEF-RedStar2[4] and fused with the appropriate pZP-promoter-terminator constructs amplified from the plasmid constructed in last step using the indicated primer pairs (Supplementary file 1, Table S3) to form different pZP-promoter-reporter-terminator constructs. For *Y. lipolytica* transformation, vectors for the expression of *RedStar2* under the control of different promoters and terminators were digested using *Not*I, thus generating a linear DNA with Zeta sequences at both extremities. Then the gel

purified expression cassettes were introduced into the Zeta docking platform of *Y. lipolytica* JMY1212 Zeta strain[3] by homologous recombination using the lithium acetate method[8]. The successful integration of the heterologous gene into the genome of *Y. lipolytica* was verified by PCR using gene specific primers.

(ii) Construction of expression cassettes for xylose assimilation pathway. The *XYL1* gene encoding NAD(P)H-dependent D-xylose reductase (XR) (GenBank accession number: XM_001385144.1), and *XYL2* gene encoding xylitol dehydrogenase (XDH) (GenBank accession number: XM_001386945.1), were amplified from gDNA of *Scheffersomyces stipitis* (ATCC® 58,785™) by PCR using primers XYL1F/XYL1R and XYL2F/XYL2R, respectively. Additionally, the *XKS1* gene encoding xylulokinase (XKS) (GenBank accession number: XM_505266.1) was amplified from gDNA of *Y. lipolytica* Po1d by PCR using primers XKS1F/XKS1R. A 15-base pair sequence homologous to the target plasmid was introduced at the 3′ and 5′ ends of each gene during PCR amplification. Then, the DNA fragment of *XYL1*, *XYL2* and *XKS1* was fused with the PCR fragment of plasmid pZP-TDH (primer pair TdhF2/TdhR2), pZP-EXP (primer pair ExpF2/ExpR2) and pZP-FBA (primer pair FbaF2/FbaR2), respectively.

(iii) Construction of expression cassettes for cellobiose assimilation pathway. Sequences encoding *Clostridium thermocellum* cellobiose phosphorylase (*CtCBP1*, GenBank accession number: YP_001036707), and *Neurospora crassa* cellodextrin transporter (*NcCDT1*, GenBank accession number: NCU00801) were synthesized by GenScript USA (Piscataway, NJ), introducing optimal codon usage features for *Y. lipolytica*, and cloned into the plasmid JMP62UraTEF and/or JMP62Urahp4d[5],

procuring plasmids JMP62UraTCbp1 and JMP62UrahCdt1, respectively. Then, the LIP2 terminator present in plasmid JMP62UraTCbp1 was replaced by the cyc1 terminator (tCYC1). This was achieved by fusing the cyc1 terminator, amplified from the gDNA of *Y. lipolytica* by PCR using primers cyc1tF/cyc1tR, with the PCR fragment amplified from the plasmid JMP62UraTCbp1 (primers JmpCBPF/JmpCBPR), using In-Fusion® HD Cloning Kit. The genes *PGM1* (YALI0E02244g), *PGM2* (YALI0E02090g) and *PCM1* (YALI0E29579g) encoding putative phosphoglucomutases in *Y. lipolytica*, were amplified from gDNA of *Y. lipolytica* Po1d by PCR using primers ylPGM1F/ylPGM1R, ylPGM2F/ylPGM2R and ylPCM1F/ylPCM1R, respectively. Additionally, the genes *PGM1* (GenBank accession number: NM_001179693.1) and *PGM2* (GenBank accession number: NM_001182605.1), encoding characterized phosphoglucomutase, were amplified from gDNA of *S. cerevisiae* by PCR using primers scPGM1F/scPGM1R and scPGM2F/scPGM2R, respectively. 15-base pair sequences homologous to the target plasmid were introduced at the 3′ and 5′ ends of each gene during PCR amplification. Then, the *ylPGM1*, *ylPGM2, ylPCM1*, *scPGM1* and *scPGM2* gene was fused with the PCR fragment of plasmid pZP-GUT (primer pair GutF2/GutR2), respectively. After construction, all expression vectors were verified by DNA sequencing (GATC Biotech, Konstanz, Germany).

Supplementary References

1. Blazeck, J., Liu, L., Redden, H. & Alper, H. Tuning gene expression in Yarrowia lipolytica by a hybrid promoter approach. *Appl Environ Microbiol* **77**, 7905-7914 (2011).

2. Nicaud, J.-M. et al. Protein expression and secretion in the yeast Yarrowia lipolytica. *FEMS Yeast Research* **2**, 371-379 (2002).
3. Bordes, F., Fudalej, F., Dossat, V., Nicaud, J.M. & Marty, A. A new recombinant protein expression system for high-throughput screening in the yeast Yarrowia lipolytica. *Journal of microbiological methods* **70**, 493-502 (2007).
4. Kabran, P., Rossignol, T., Gaillardin, C., Nicaud, J.M. & Neuveglise, C. Alternative splicing regulates targeting of malate dehydrogenase in Yarrowia lipolytica. *DNA Res* **19**, 231-244 (2012).
5. Madzak, C., Tréton, B.Y. & Blanchin-Roland, S. Strong hybrid promoters and integrative expression/secretion vectors for quasi-constitutive expression of heterologous proteins in the yeast Yarrowia lipolytica. *Journal of molecular microbiology and biotechnology* **2 2**, 207-216 (2000).
6. Vernis, L. et al. An origin of replication and a centromere are both needed to establish a replicative plasmid in the yeast Yarrowia lipolytica. *Molecular and cellular biology* **17**, 1995-2004 (1997).
7. Guo, Z.P. et al. Developing cellulolytic Yarrowia lipolytica as a platform for the production of valuable products in consolidated bioprocessing of cellulose. *Biotechnol Biofuels* **11**, 141 (2018).
8. Duquesne, S., Bordes, F., Fudalej, F., Nicaud, J.-M. & Marty, A. in Lipases and Phospholipases: Methods and Protocols. (ed. G. Sandoval) 301-312 (Humana Press, Totowa, NJ; 2012).
