# Supplementary material for: An artificial chromosome ylAC enables efficient assembly of multiple genes in Yarrowia lipolytica for biomanufacturing
Source: Commun Biol. 2020 Apr 29;3:199. doi: 10.1038/s42003-020-0936-y (PMC7190667; doi:10.1038/s42003-020-0936-y)
Supplement: Supplementary file 2 — Description of Additional Supplementary Files [file 42003_2020_936_MOESM2_ESM.pdf]

Description of Additional Supplementary Files

File Name: **Supplementary Data 1**
Description: Sequences of oligonucleotides used in this study

File Name: **Supplementary Data 2**
Description: All source data underlying the tables, and the graphs and charts presented in the main figures
